# Does "ichthyosis uteri" have malignant potential? : A case report of squamous cell carcinoma of endometrium associated with extensive ichthyosis uteri

**DOI:** 10.1186/1746-1596-3-4

**Published:** 2008-01-31

**Authors:** Kanchan Murhekar, Urmila Majhi, V Sridevi, T Rajkumar

**Affiliations:** 1 Department of Pathology, Cancer Institute, Adyar, Chennai, India; 2 Department of Surgical Oncology, Cancer Institute, Adyar, Chennai, India; 3 Department of Molecular Oncology, Cancer Institute, Adyar, Chennai, India

## Abstract

Ichthyosis uteri is a rare condition in which the entire surface of the endometrium is replaced by stratified squamous epithelium. Though the condition often is considered as benign, anaplastic and dysplastic changes have been reported. We describe herein a rare case of low-grade squamous cell carcinoma of endometrium associated with extensive ichthyosis uteri with dysplasia. The cervix showed moderate to severe dysplastic changes while the right fallopian tube showed extensive squamous metaplasia with dysplastic changes. We conclude that squamous cell carcinoma could develop into pre-existing ichthyosis uteri.

## Clinical History

A 65-year-old multi-gravida presented with complaints of abdominal pain and postmenopausal bleeding per vagina for seven months. Her past medical history was insignificant with no history of tuberculosis, inflammatory conditions of the uterus or iatrogenically introduced substances in uterus. She had attained menopause about 15 years back.

Gynecological examination revealed atrophic ectocervix flushed with vagina. The vagina appeared normal. Uterus was bulky of around 12 weeks and adnexae were unremarkable. Colposcopy showed schiller's unstained areas on anterior lip of cervix. Ultrasound abdomen showed endometrial thickness of 2.6 cms. and fluid in the endometrial cavity. Ectocervical biopsy showed strips of moderate to severe dysplastic stratified squamous epithelium. Endometrial curetting revealed strips of stratified squamous epithelium showing moderate dysplastic changes. No normal endometrium was seen. The pyometra was drained, following which the patient underwent type-II radical hysterectomy. The procedure was well tolerated and the postoperative period was uneventful.

## Pathologic findings

### Macroscopic

The hysterectomy specimen revealed thickened and widened endometrial cavity with gray white nodule in the sub-adjacent myometrium. The cervix showed no obvious growth. The cut-section of the right fallopian tube showed thickened mucosa. Both the ovaries did not show any gross abnormality.

### Microscopic

**Figure 1:**
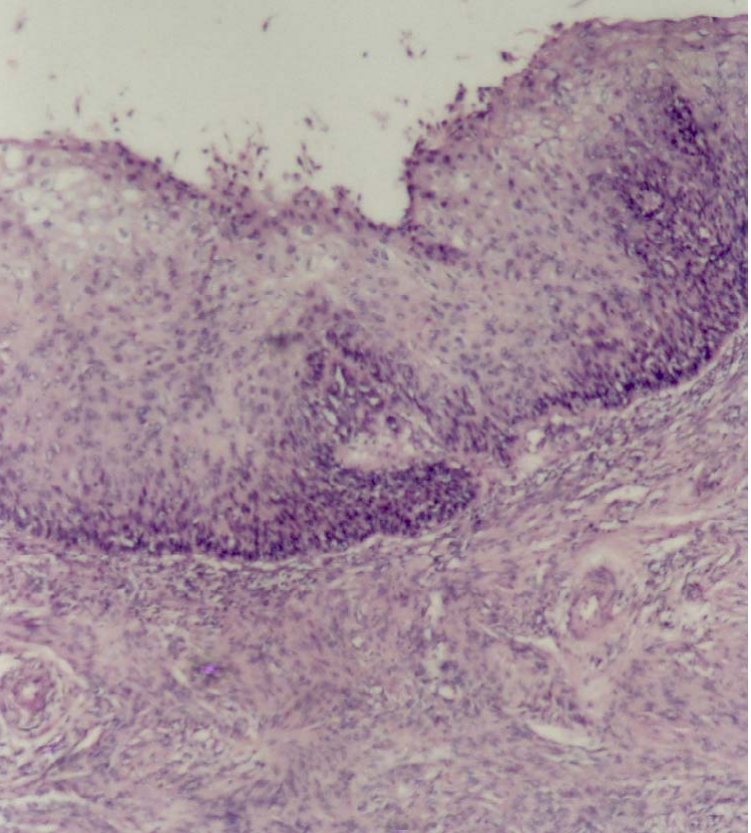
Extensive squamous metaplasia of endometrium with dysplastic changes overlying myometrial stroma.

**Figure 2:**
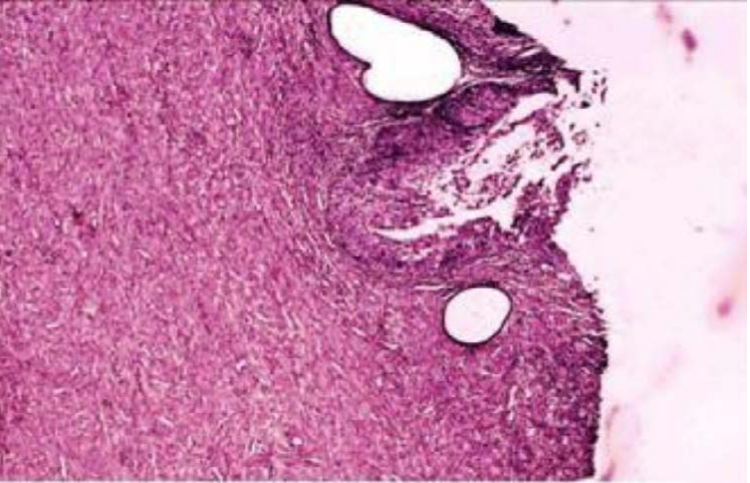
Squamous metaplasia of endometrium with few atrophic endometrial glands.

**Figure 3:**
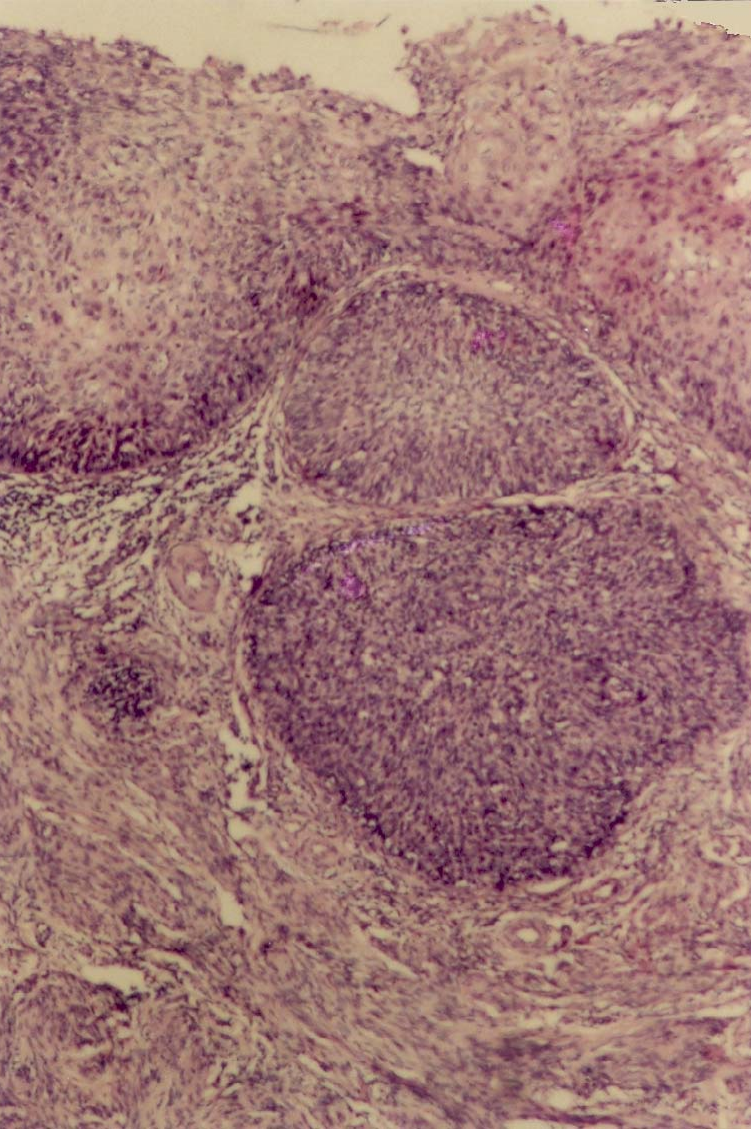
Invasive islands of atypical squamous cells in the myometrium with a background of plaque-like squamous epithelium.

**Figure 4:**
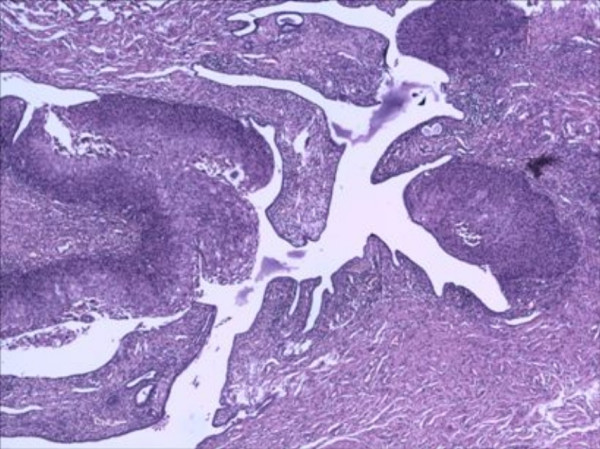
Extensive squamous metaplasia with dysplastic changes of fallopian tube.

The sections revealed entire endometrium replaced by stratified squamous epithelium showing areas of heavy keratinization, koilocytic changes, nuclear hyperchromasia and moderate increase in nuclear-cytoplasmic ratio indicating low grade dysplastic changes in underlying ichthyosis uteri (Fig 1). Extensive sampling of endometrium revealed few atrophic endometrial glands beneath the dysplastic squamous epithelium (Fig 2). Focal invasive islands of atypical squamous epithelial cells were seen in superficial myometrium (Fig 3). In addition, myometrium showed few foci of adenomyosis. Section from the cervix showed moderate to severe dysplastic changes with extension into endocervical glands. The right fallopian tube showed extensive squamous metaplasia with moderate to severe dysplastic changes (fig 4).

## Discussion

Zeller coined the term "ichthyosis uteri" in the year 1885 to describe a condition of extensive squamous metaplasia of the surface endometrium following iatrogenically introduced caustic substances such as formalin or iodine [1]. The condition is exceedingly rare with few cases reported in the literature. The case that we report here showed low-grade squamous cell carcinoma of endometrium in pre-existing extensive ichthyosis uteri with dysplasia. The cervix showed moderate to severe dysplastic changes while the right fallopian tube showed extensive squamous metaplasia with dysplastic changes. The most likely explanation of these composite findings is that the squamous cell carcinoma of the endometrium developed within the background of extensive ichthyosis uteri. Factors in favor of this explanation are (1) thickened endometrium showing dysplastic to low grade squamous intraepithelial lesion with areas of invasion into myometrium (2) No obvious growth in cervix and (3) post menopausal age of the patient. The squamous metaplasia in one of the fallopian tube could be on account of proximal extension from extensive ichthyosis in the endometrium.

Ichthyosis is considered as benign condition. However, anaplastic and dysplastic changes have been reported by some authors [1-3]. These cases however were associated with dysplasia or carcinoma of the cervix, and authors argued for direct extension from cervical pathology for the findings in endometrium. The case described by Patton and Squares [1] had extensive high-grade dysplasia of the cervix (with a focal area of micro-invasion) and ichthyosis uteri of the endometrium. The areas of the squamous epithelium in the endometrium showed some degree of "cellular anaplasia". The authors interpreted this finding as a direct extension from the cervix. Pins et al described a case in which high-grade dysplasia of the cervix (and without invasion) extended proximally and coated the entire endometrium [2]. Recently Fadare [3] reported a case of moderately differentiated squamous cell carcinoma of uterine cervix associated with extensive ichthyosis uteri like changes of the entire endometrium that additionally, had superimposed low-grade dysplastic changes. The patient also had squamous cell carcinoma of cervix and was positive for human papilloma virus (HPV) infection on immunohistochemical staining. The most plausible explanation for these findings was that a squamous cell carcinoma of the cervix extended proximally and colonized the pre-existing ichthyosis uteri by associated HPV [3]. Bewtra et al [4] described a case of extensive benign squamous keratinization with underlying endometrial adenocarcinoma.

Besides the dysplastic or anaplastic changes, a case of malignant degeneration has also been reported in ichthyosis uteri [5]. This case presented as a squamous cell carcinoma of the corpus uteri at the base of ichthyosis uteri. The ichthyosis uteri developed over a period of just three years from a discrete squamous cell metaplasia of the endometrial body in the presence of slight nonspecific endometritis. Our case closely resembles the case described by Heckeroth and Ziegler [5].

In summary, we describe a case of low-grade squamous cell carcinoma of endometrium developing in pre-existing extensive ichthyosis uteri with dysplasia. Though considered as benign condition, based on the present case report and that of Heckeroth et al [5], the possibility of malignant degeneration in pre-existing ichthyosis uteri cannot be completely ruled out.

## Competing interests

The author(s) declare that they have no competing interests.

## Authors' contributions

KM, UM, VS drafted the manuscript. TR revised the draft critically for intellectual content. All authors read and approved the final manuscript.
